# Comparison of six antibody assays and two combination assays for COVID-19

**DOI:** 10.1186/s12985-022-01752-y

**Published:** 2022-02-03

**Authors:** Masaki Yamamoto, Kazuyuki Okazaki, Yoko Kitai, Koh Shinohara, Satomi Yukawa, Taro Noguchi, Michio Tanaka, Yasufumi Matsumura, Yukiko Nishiyama, Miki Nagao

**Affiliations:** 1 Department of Clinical Laboratory Medicine, Kyoto University Graduate School of Medicine, 54 Shogoin-Kawaharacho, Sakyo-ku, Kyoto, 606-8507 Japan; 2 Department of Clinical Laboratory, Kyoto University Hospital, 54 Shogoin-Kawaharacho, Sakyo-ku, Kyoto, 606-8507 Japan

**Keywords:** COVID-19, SARS-CoV-2, Antibody testing, Serology

## Abstract

**Introduction:**

In this work, six SARS-CoV-2-specific antibody assays were evaluated, namely, two pan-immunoglobulin (pan-Ig) assays [Roche Elecsys Anti-SARS-CoV-2 (named "Elecsys" in this study) and the PerkinElmer SuperFlex™ Anti-SARS-CoV-2 Ab Assay (SuperFlex_Ab)], two IgM assays [SuperFlex™ Anti-SARS-CoV-2 IgM Assay (SuperFlex_IgM) and YHLO iFlash-SARS-CoV-2 IgM (iFlash_IgM)], and two IgG assays [SuperFlex™ Anti-SARS-CoV-2 IgG Assay (SuperFlex_IgG) and iFlash-SARS-CoV-2 IgG (iFlash_IgG)]. Combination assays of SuperFlex™ (SuperFlex_any) and iFlash (iFlash_any) were also evaluated.

**Methods:**

A total of 438 residual serum samples from 54 COVID-19 patients in the COVID-19 group and 100 samples from individuals without evidence of SARS-CoV-2 infection in the negative control group were evaluated.

**Results:**

In the early stage of COVID-19 infection, within 14 days of symptom onset, the seropositive rate was lower than that of the late stage 15 days after onset (65.4% vs 99.6%). In the total period, the pan-Ig and IgG assays had higher sensitivity (90.8–95.3%) than the IgM assays (36.5–40.7%). SuperFlex_Ab and SuperFlex_any had higher sensitivity than Elecsys and SuperFlex_IgG (p < 0.05). The specificity of all the assays was 100%, except for SuperFlex_IgM (99.0%). The concordance rate between each assay was higher (96.4–100%) in the late stage than in the early stage (77.4–98.1%).

**Conclusion:**

For the purpose of COVID-19 diagnosis, antibody testing should be performed 15 days after onset. For the purpose of epidemiological surveillance, highly sensitive assays should be used as much as possible, such as SuperFlex_Ab, iFlash_IgG and their combination. IgM assays were not suitable for these purposes.

## Introduction

Since the start of the coronavirus disease 2019 (COVID-19) pandemic and its worldwide spread, the gold standard for diagnosis has been the identification of viral RNA by reverse-transcription polymerase chain reaction (RT-PCR) from nasal swabs, nasopharyngeal swabs, or saliva [1]. RT-PCR can detect the infectious severe acute respiratory syndrome coronavirus (SARS-CoV-2) virus itself with high sensitivity and specificity. However, false-negative results can be obtained due to sampling errors, the sample collection site, swab type, timing of sample collection, skill of the laboratory staff, etc. [2].

Antibody testing for COVID-19 can be a complementary diagnostic tool to RT-PCR. Because seroconversion is generally observed 3 to 14 days after symptom onset, antibody testing is not suitable for the early diagnosis of COVID-19 [3]. However, it can be suitable for the following purposes: (1) the diagnosis of patients more than 7 days after symptom onset, (2) the diagnosis of patients with a negative RT-PCR test but with strong suspicion of COVID-19 infection, (3) contact tracing, (4) the determination of potential immunity, and (5) serosurveillance [4]. At present, many antibody assays for COVID-19 are available and have been authorized by the U.S. Food and Drug Administration (FDA) under emergency use authorization (EUA) [5]. Although these assays have good sensitivity and specificity (87.9–100% sensitivity and 95.0–100% specificity for IgG), the sensitivity and specificity values are mainly determined 14 days from symptom onset or later. For the purpose of serosurveillance, it is desirable to select an antibody test that has good sensitivity and specificity not only for the late stage of COVID-19 infection but also the early stage so that seropositive cases of early-stage COVID-19 can be detected. Thus, we compared six assays and two combination assays, including one assay approved under EUA by the FDA, with the goal of identifying the assay(s) with the highest sensitivity and specificity.

## Materials and methods

### Study settings

Sera from two distinct groups, the COVID-19 group (COG) and the negative control group (NCG), were analyzed in this study. The COG samples were composed of a total of 438 residual serum samples from 54 COVID-19 patients who were admitted to Kyoto University Hospital, Kyoto, Japan from April 2020 to January 2021. All patients were confirmed to have COVID-19 infection by RT-PCR using saliva and/or nasopharyngeal swab samples. All patients were confirmed to have COVID-19 infection by RT-PCR using saliva and/or nasopharyngeal swab samples, which were performed because individuals had symptoms of COVID-19 or were in close contact with COVID-19 patients [6]. The characteristics of these patients are shown in Table 1. The numbers of patients and samples and the timing of sample collection are shown in Table 2. The severity of COVID-19 was defined according to the World Health Organization (WHO) severity classification [7].

**Table 1:** Demographic characteristics of patients in COVID-19 group

	Total COVID-19 patients (n = 54)	Seropositive patients* (n = 48)	Seronegative patients (n = 6)	p value
Age, years old (IQR)	69.5 (57.25–82.5)	71 (57.75–83.5)	65.5 (57.3–73.8)	0.31
Sex, female (%)	13 (24.1%)	10 (20.8%)	5 (50.0%)	0.14
*Severity, no. (%)*				< 0.01
Critical	21 (38.9%)	21 (43.8%)	0 (0%)	0.07
Severe	17 (31.5%)	16 (33.3%)	1 (16.7%)	0.65
Moderate	9 (16.7%)	7 (14.6%)	2 (33.3%)	0.26
Mild	5 (9.3%)	2 (4.2%)	3 (50.0%)	< 0.01
Asymptomatic	2 (3.7%)	2 (4.2%)	0 (0%)	1.00
*Respiratory support, no. (%)*				< 0.01
MV and ECMO	5 (9.3%)	5 (10.4%)	0 (0%)	1.00
MV	14 (25.9%)	14 (29.2%)	0 (0%)	0.32
None or oxygen	35 (64.8%)	29 (60.4%)	6 (100%)	0.08
*Administration*				
Collected samples, no. (IQR)	5 (3–9)	7 (3–10.25)	2 (2–2.75)	< 0.01
Days of serum sample collection from onset, median days (IQR)	18 (12–39)	19 (12–40)	6 (3–10)	< 0.01

IQR, interquartile range; NA, data not available; MV, mechanical ventilation; ECMO, extra corporeal membrane oxygenation; ICU, intensive care unit

*Patients who were positive for at least one of SARS-CoV-2 specific antibody testing

**Table 2:** Patients and Samples analyzed in this study, and result of each assay in each period

Days from symptom onset	Total patients, no	Seropositive patients*, no. (%)	Total collected samples, no	Samples with positive result**, no. (%)	Elecsys, no. (%)	SuperFlex_Ab, no. (%)	SuperFlex_IgM, no. (%)	SuperFlex_IgG, no. (%)	SuperFlex_any†, no. (%)	iFlash_IgM, no. (%)	iFlash_IgG, no. (%)	iFlash_any^#^, no. (%)
Day 1–7	23	14 (60.9%)	53	24 (45.3%)	16 (30.2%)	18 (34.0%)	3 (5.7%)	11 (20.8%)	18 (34.0%)	8 (15.1%)	19 (35.8%)	19 (35.8%)
Day 8–14	39	32 (82.1%)	109	82 (75.2%)	66 (60.6%)	69 (63.3%)	18 (16.5%)	61 (56.0%)	71 (65.1%)	34 (31.2%)	71 (65.1%)	71 (65.1%)
Day 1–14 total	62	46 (74.2%)	162	106 (65.4%)	82 (50.6%)	87 (53.7%)	21 (13.0%)	72 (44.4%)	89 (54.9%)	42 (25.9%)	90 (55.6%)	90 (55.6%)
Day 15–21	29	29 (100%)	78	77 (98.7%)	76 (97.4%)	76 (97.4%)	47 (60.3%)	76 (97.4%)	76 (97.4%)	50 (64.1%)	77 (98.7%)	77 (98.7%)
Day 22–28	19	19 (100%)	45	45 (100%)	45 (100%)	45 (100%)	33 (73.3%)	45 (100%)	45 (100%)	37 (82.2%)	45 (100%)	45 (100%)
Day 29–56	15	15 (100%)	80	80 (100%)	74 (92.5%)	80 (100%)	35 (43.8%)	80 (100%)	80 (100%)	25 (31.3%)	80 (100%)	80 (100%)
Day 57–84	6	6 (100%)	20	20 (100%)	20 (100%)	20 (100%)	3 (15.0%)	20 (100%)	20 (100%)	1 (5.0%)	20 (100%)	20 (100%)
Day 85–112	1	1 (100%)	12	12 (100%)	12 (100%)	12 (100%)	0 (0%)	12 (100%)	12 (100%)	0 (0%)	12 (100%)	12 (100%)
Day 113–140	2	2 (100%)	14	14 (100%)	14 (100%)	14 (100%)	0 (0%)	14 (100%)	14 (100%)	0 (0%)	14 (100%)	14 (100%)
Day 141–182	2	2 (100%)	27	27 (100%)	25 (92.6%)	27 (100%)	0 (0%)	27 (100%)	27 (100%)	0 (0%)	22 (81.5%)	22 (81.5%)
Day 15–182 total	74	74 (100%)	276	275 (99.6%)	266 (96.4%)	274 (99.3%)	118 (42.8%)	274 (99.3%)	274 (99.3%)	113 (40.9%)	270 (97.8%)	270 (97.8%)
Total	136	120 (88.2%)	438	381 (87.0%)	348 (79.5%)	361 (82.4%)	139 (31.7%)	346 (79.0%)	363 (82.9%)	155 (35.4%)	360 (82.2%)	360 (82.2%)

*Patients who were positive for at least one of SARS-CoV-2 specific antibody testing

**Samples with positive result for at least one of SARS-CoV-2 specific antibody testing

^†^The SuperFlex_any is the assay combined with SuperFlex_Ab, SuperFlex_IgM, and SuperFlex_IgG

^#^The iFlash_any is the assay combined with iFlash_IgM and iFlash_IgG

The NCG samples included 100 of 1589 randomly selected serum samples that were derived from regional epidemiological surveillance of COVID-19 from September 2020 to October 2020 in Kyoto City, Japan. None of the NCG group participants tested positive by RT-PCR or had evidence of COVID-19 infection.

### Laboratory methods and antibody testing

Sera from patients and research participants were separated after centrifugation and stored at − 80 °C in a deep freezer until analysis. The samples were analyzed using six assays from three manufacturers according to the manufacturer’s instructions, including calibration and quality control (QC) (Table 3). QC tests of each assay were performed using QC reagents supplied by each company [reciControl Anti-SARS-CoV-2 for Elecsys, provided QC reagents for SuperFlex (control 1 and control 2 are provided each assay kit), and 2019-nCovIgG Control 2019-nCovIgM Control for iFlash]. These assays consisted of two pan-immunoglobulin (pan-Ig) assays [Roche Elecsys Anti-SARS-CoV-2 (named “Elecsys” in this study) and PerkinElmer SuperFlex™ Anti-SARS-CoV-2 Ab Assay (SuperFlex_Ab)], two IgM assays [SuperFlex™ Anti-SARS-CoV-2 IgM Assay (SuperFlex_IgM) and YHLO iFlash-SARS-CoV-2 IgM (iFlash_IgM)], and two IgG assays [SuperFlex™ Anti-SARS-CoV-2 IgG Assay (SuperFlex_IgG) and iFlash-SARS-CoV-2 IgG (iFlash_IgG)]. Elecsys is approved under EUA by the US FDA and marked with CE-in vitro-diagnostic medical devices (IVDs), and it has 100% sensitivity [95% confidence interval (CI): 88.3–100%] and 99.8% specificity (95% CI: 99.7–99.9%) [5]. SuperFlex_IgG, iFlash_IgM, and iFlash_IgG are also marked with CE-IVD. Information about the measurement method and target antigens of these assays is also shown in Table 3. Furthermore, we evaluated two combination assays: “SuperFlex_any”, which is a combination of SuperFlex_Ab, SuperFlex_IgM, and SuperFlex_IgG, and “iFlash_any”, which is an iFlash_IgM and iFlash_IgG combination. At present, there is no standard method available for SARS-CoV-2 antibody testing for diagnosis and serosurveillance. Therefore, if seroconversion was observed and at least one of these assays was positive, SARS-CoV-2 antibody testing was defined as seropositive in this study.

**Table 3:** Assays for SARS-CoV-2 antibody testing used in this study

	Assay name (in this study)	Manufacturer (using instrument)	Measurement method	Target protein	Immunoglobulin class	FDA EUA	CE-IVD
1	Elecsys Anti-SARS-CoV-2 (Elecsys)	Roche (Cobas e602)	ECLIA	N	Pan-Ig	(+)	(+)
2	SuperFlex™ Anti-SARS-CoV-2 Ab Assay (SuperFlex_Ab)	PerkinElmer (SuperFlex)	CLIA	S	Pan-Ig	(−)	(−)
3	SuperFlex™ Anti-SARS-CoV-2 IgM Assay (SuperFlex_IgM)	PerkinElmer (SuperFlex)	CLIA	N and S	IgM	(−)	(−)
4	SuperFlex™ Anti-SARS-CoV-2 IgG Assay (SuperFlex_IgG)	PerkinElmer (SuperFlex)	CLIA	S	IgG	(−)	(+)
5	iFlash-SARS-CoV-2 IgM (iFlash_IgM)	Shenzhen YHLO (iFlash 3000)	CLIA	N and (S)	IgM	(−)	(+)
6	iFlash-SARS-CoV-2 IgG (iFlash_IgG)	Shenzhen YHLO (iFlash 3000)	CLIA	N and (S)	IgG	(−)	(+)

FDA, Food and Drug Administration; EUA, Emergency Use Authoraization; ECLIA, electro chemiluminescence immunoassay; CLIA, chemiluminescent immunoassay; N, nucleocapsid protein; S, spike protein; Pan-Ig, Pan-immunogulobulin

### Statistical analysis

All statistical analyses were performed using R version 4.0.3 [8]. A p-value of < 0.05 was considered statistically significant. Fisher's exact test was used to compare categorical variables. Mann–Whitney U-test was used to compare ordinal or continuous variables. McNemar’s exact test was used to compare the sensitivities between each assay. To observe agreement, the agreement rate, Cohen’s kappa, and Gwet’s AC1 were calculated between each assay, with the exception of IgM assays, which have obviously lower positivity rates than the other assays [9].

## Results

### Patients and samples

Among the 54 patients in the COG, the median age was 69.5 years old [interquartile range (IQR) 57.25–82.5], and 24.1% were female (Table 1). Most of these patients were seriously ill, as follows: 38.9% were in a critical state, and 31.5% were in a severe state. Both mechanical ventilation (MV) and extracorporeal membrane oxygenation (ECMO) were used in 9.3% of patients, and MV without ECMO was used in 25.9%. Seroconversion was observed in 48 (88.9%) of 54 patients, who were defined as seropositive patients (Table 1). During the study period, a total of 438 serum samples were analyzed, among which 381 samples (87.0%) were positive in at least one of the COVID-19 antibody assays. The NCG included 100 randomly selected participants from 1589 regional epidemiological studies. They were essential workers in Kyoto City, Japan [median age: 43 years old (IQR 33–49.5), female: 41%]. All their SARS-CoV-2 RT-PCR tests were negative, and they had no evidence of COVID-19 infection, such as clinical manifestations and close contact with COVID-19 patients.

### Positivity rate among serum samples of COVID-19 patients

**Fig. 1:**
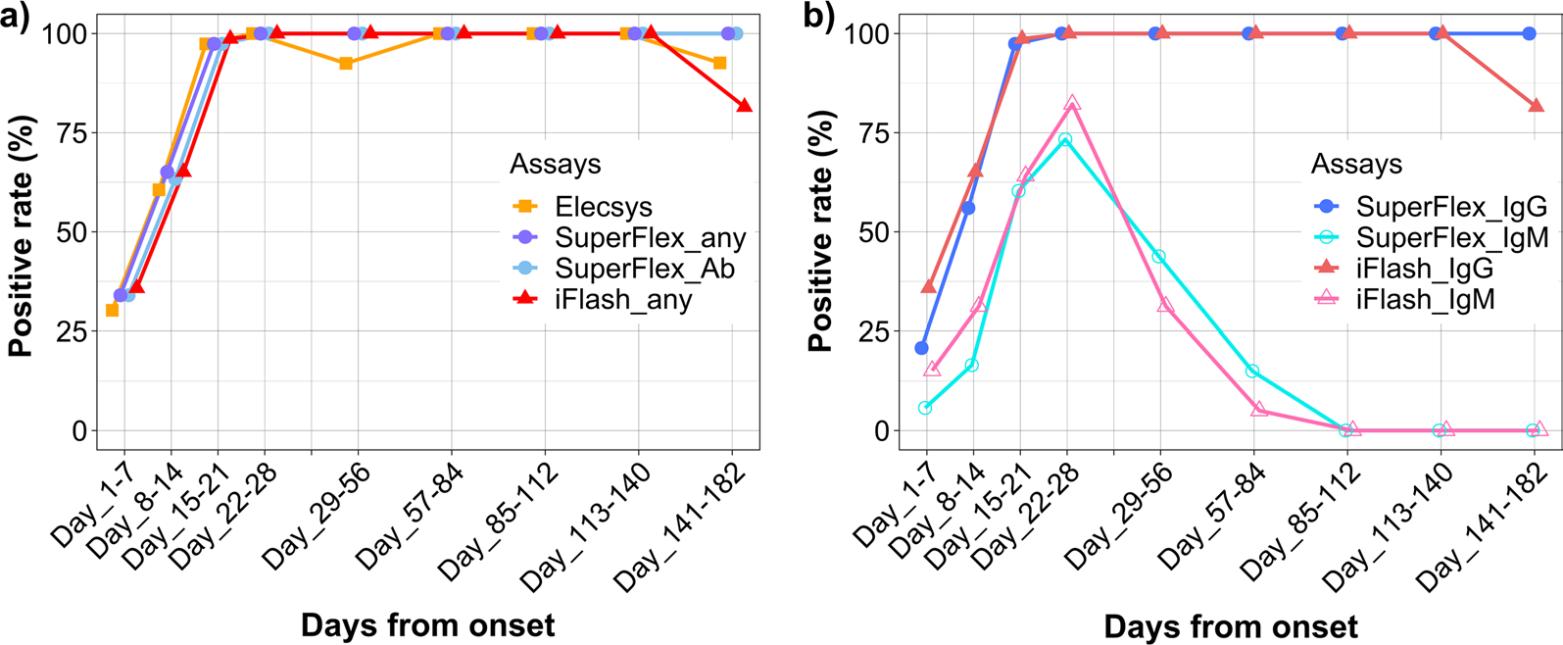
The positivity rate of each assay on each day from symptom onset. **a** The positivity rate of the pan-immunoglobulin assays [Elecsys (orange line) and SuperFlex_Ab (cyan line)] and combination assays [SuperFlex_any (purple line) and iFlash_any (red line)]. **b** The positivity rate of the IgM assays [SuperFlex_IgM (aqua line) and iFlash_IgM (pink line)] and IgG assays [SuperFlex_IgG (purple line) and iFlash_IgG (red line)]

The positivity rate of antibody testing among serum samples of the COG varied according to the days after symptom onset and the assay (Fig. 1). In the early stage of COVID-19, within 14 days of symptom onset, the positivity rate was lower than that in the late stage, at 15 days after onset (65.4% vs 99.6%). The IgM assays had lower positivity rates than the other assays, including the pan-Ig, IgG, and combination assays. The highest positivity rates among the IgM assays occurred between day 22 and day 28 after onset, at 76.7% (SuperFlex_IgM) and 86.0% (iFlash_IgM). Fifteen days after onset, almost all pan-Ig and IgG assays revealed positive results (92.5–100%).

### Sensitivity, specificity, positive predictive value, and negative predictive value

**Fig. 2:**
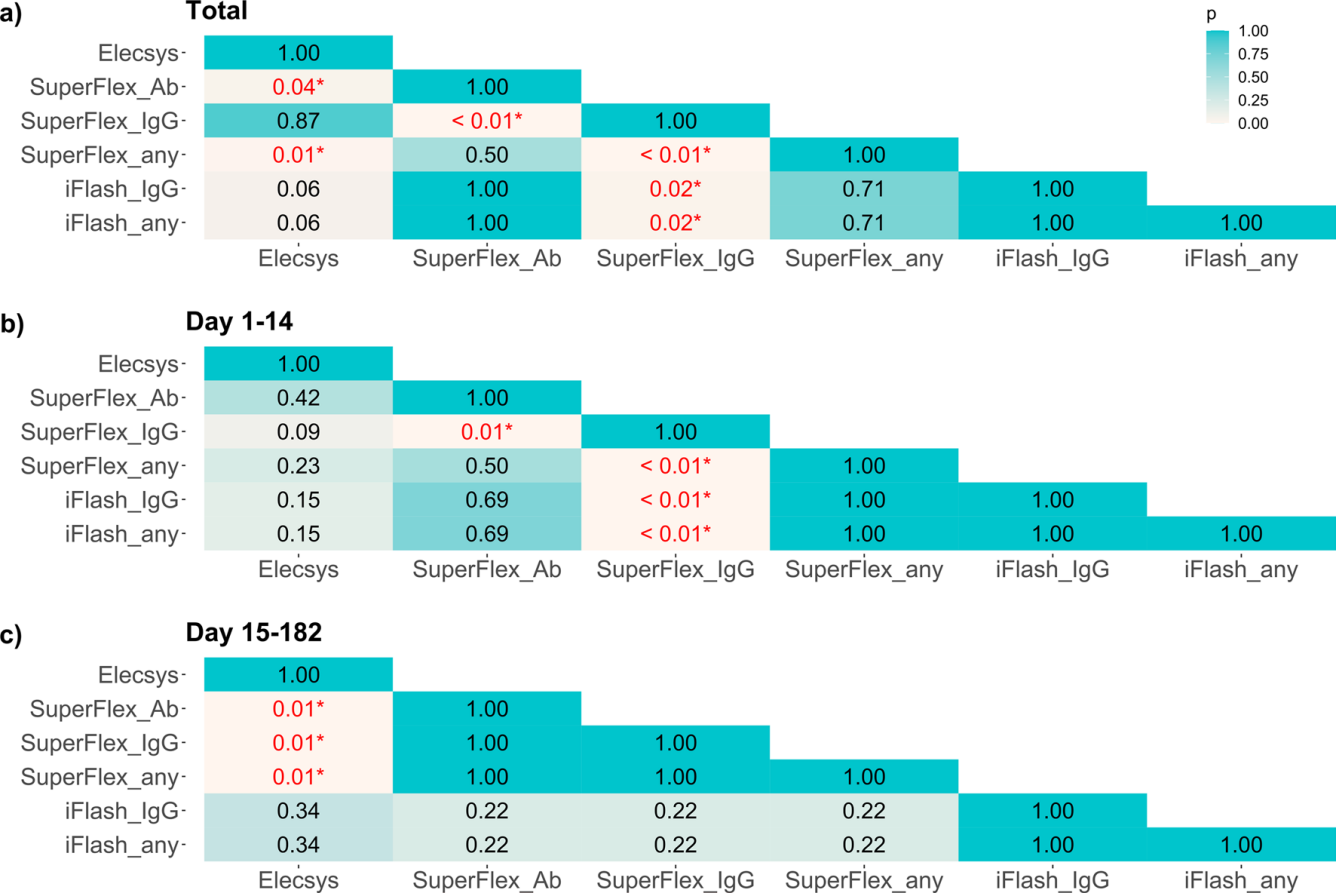
McNemar's exact test was used to compare each assay (pan-Ig assays and IgG assays). An asterisk indicates significantly different performance

**Fig. 3:**
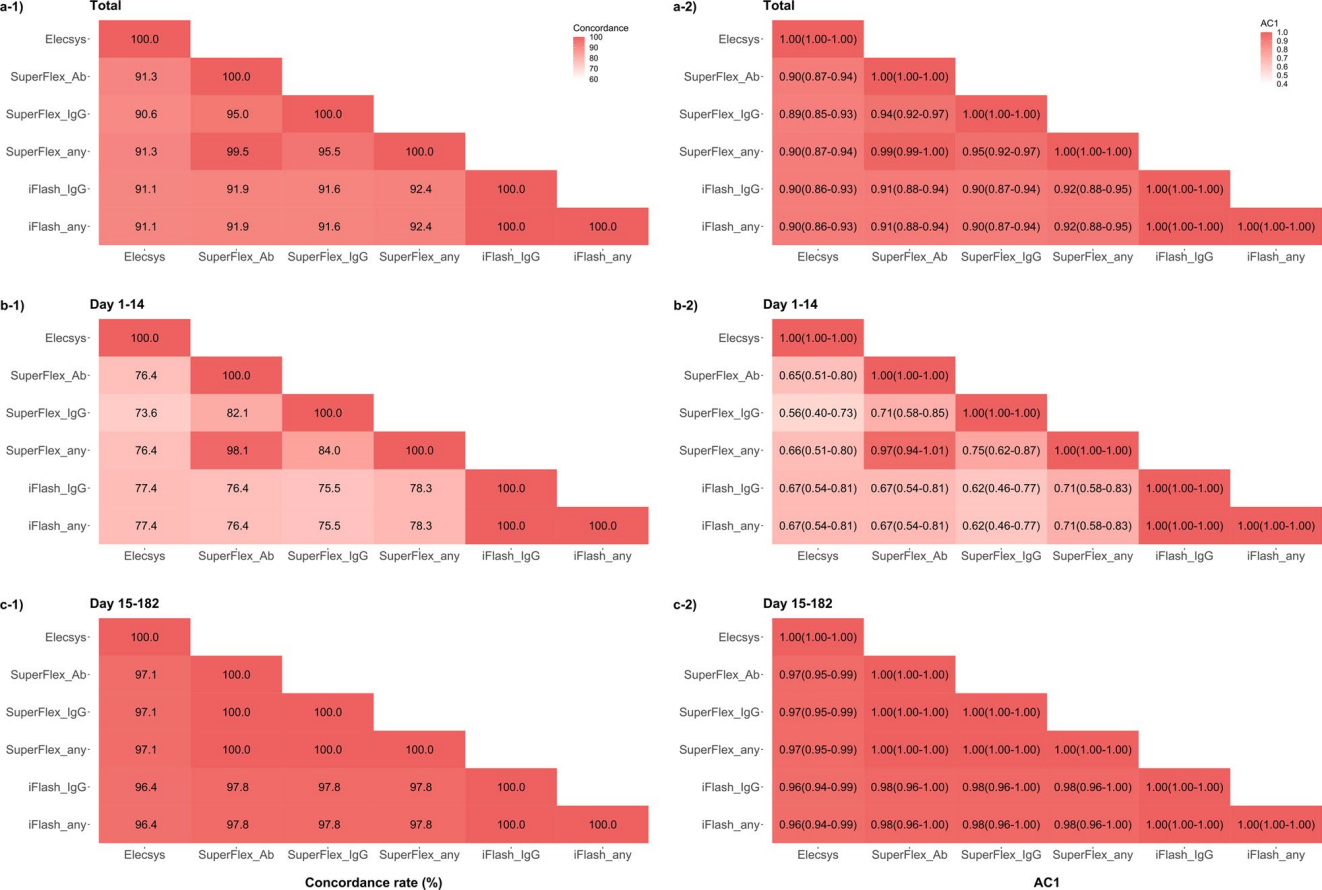
The agreement of each assay using the concordance rate and Gwet's AC1. **a-1**–**c-1** show the results of the concordance rate between each assay in each period. **a-2**–**c-2** show the results of Gwet's AC1 (95% confidence interval)

The sensitivity, specificity, positive predictive value (PPV), and negative predictive value (NPV) of the assays are shown in Table 4. In general, the pan-Ig, IgG, and combination assays had good sensitivities, specificities, PPVs and NPVs. Similar to the positivity rate, the sensitivities in the early stage were lower than those in the late stage. In each stage, IgM assays had lower sensitivities than other assays. Then, we compared pan-Ig assays (Elecsys and SuperFlex_Ab), IgG assays (SuperFlex_IgG and iFlash_IgG), and combination assays (SuperFlex_any and iFlash_any). In the total period, SuperFlex_Ab and SuperFlex_any had higher sensitivities than Elecsys and SuperFlex_IgG (p < 0.05, Fig. 2a)). iFlash_IgG and iFlash_any also had higher sensitivities than SuperFlex_IgG (p = 0.02). The specificity of all the assays was 100%, except for SuperFlex_IgM (99.0%), and this result affected the specificity of SuperFlex_any. In the early stage, SuperFlex_Ab, SuperFlex_any, iFlash_IgG, and iFlash_any had relatively higher sensitivity (82.1–84.9%). In the late stage, the concordance rate between each assay was high (96.4–100%, Fig. 3), and the results of Gwet’s AC1 also supported this finding. However, in the early stage, the concordance rate between each assay was likely to be low (77.4–98.1%).

**Table 4:** Sensitivity, specificity, positive predicted value, and negative predicted value

	Sensitivity (95% CI)	Specificity (95% CI)	PPV (95% CI)	NPV (95% CI)
*Total*				
Elecsys	91.3% (88.1–94.0%)	100% (96.4–100%)	100% (98.9–100%)	75.2% (67.0–82.3%)
SuperFlex_Ab	94.8% (92.0–96.8%)	100% (96.4–100%)	100% (99.0–100%)	83.3% (75.4–89.5%)
SuperFlex_IgM	36.5% (31.6–41.5%)	99.0% (94.6–100%)	99.3% (96.1–100%)	29.0% (24.3–34.2%)
SuperFlex_IgG	90.8% (87.5–93.5%)	100% (96.4–100%)	100% (98.9–100%)	74.1% (65.8–81.2%)
SuperFlex_any*	95.3% (92.6–97.2%)	99.0% (94.6–100%)	99.7% (98.5–100%)	84.6% (76.8–90.6%)
iFlash_IgM	40.7% (35.7–45.8%)	100% (96.4–100%)	100% (97.6–100%)	30.7% (25.7–36.0%)
iFlash_IgG	94.5% (91.7–96.6%)	100% (96.4–100%)	100% (99.0–100%)	82.6% (74.7–88.9%)
iFlash_any#	94.5% (91.7–96.6%)	100% (96.4–100%)	100% (99.0–100%)	82.6% (74.7–88.9%)
*Day 1–14*				
Elecsys	77.4% (68.2–84.9%)	100% (96.4–100%)	100% (95.6–100%)	80.6% (72.6–87.2%)
SuperFlex_Ab	82.1% (73.4–88.8%)	100% (96.4–100%)	100% (95.8–100%)	84.0% (76.2–90.1%)
SuperFlex_IgM	19.8% (12.7–28.7%)	99.0% (94.6–100%)	95.5% (77.2–99.9%)	53.8% (46.3–61.2%)
SuperFlex_IgG	67.9% (58.2–76.7%)	100% (96.4–100%)	100% (95.0–100%)	74.6% (66.4–81.7%)
SuperFlex_any*	84.0% (75.6–90.4%)	99.0% (94.6–100%)	98.9% (94.0–100%)	85.3% (77.6–91.2%)
iFlash_IgM	39.6% (30.3–49.6%)	100% (96.4–100%)	100% (91.6–100%)	61.0% (53.1–68.5%)
iFlash_IgG	84.9% (76.6–91.1%)	100% (96.4–100%)	100% (96.0–100%)	86.2% (78.6–91.9%)
iFlash_any#	84.9% (76.6–91.1%)	100% (96.4–100%)	100% (96.0–100%)	86.2% (78.6–91.9%)
*Day 15–182*				
Elecsys	96.7% (93.9–98.5%)	100% (96.4–100%)	100% (98.6–100%)	91.7% (84.9–96.2%)
SuperFlex_Ab	99.6% (98.0–100%)	100% (96.4–100%)	100% (98.7–100%)	99.0% (94.6–100%)
SuperFlex_IgM	42.9% (37.0–49.0%)	99.0% (94.6–100%)	99.2% (95.4–100%)	38.7% (32.7–44.9%)
SuperFlex_IgG	99.6% (98.0–100%)	100% (96.4–100%)	100% (98.7–100%)	99.0% (94.6–100%)
SuperFlex_any*	99.6% (98.0–100%)	99.0% (94.6–100%)	99.6% (98.0–100%)	99.0% (94.6–100%)
iFlash_IgM	41.1% (35.2–47.2%)	100% (96.4–100%)	100% (96.8–100%)	38.2% (32.3–44.3%)
iFlash_IgG	98.2% (95.8–99.4%)	100% (96.4–100%)	100% (98.6–100%)	95.2% (89.2–98.4%)
iFlash_any#	98.2% (95.8–99.4%)	100% (96.4–100%)	100% (98.6–100%)	95.2% (89.2–98.4%)

PPV, positive predicted value; NPV, negative predicted value; CI, confidence interval

*The SuperFlex_any is the assay combined with SuperFlex_Ab, SuperFlex_IgM, and SuperFlex_IgG

^#^ The iFlash_any is the assay combined with iFlash_IgM and iFlash_IgG

## Discussion

The six assays and two combination assays for COVID-19 antibody testing were compared using 438 serum samples from 54 confirmed COVID-19 patients and 100 samples from COVID-19-negative individuals in this study. The 2 pan-Ig assays (Elecsys and SuperFlex_Ab), two IgG assays (SuperFlex_IgG and iFlash_IgG), and two combination assays (SuperFlex_any and iFlash_any) had high sensitivities, specificities, PPVs and NPVs. Moreover, the positivity rates of these assays among COVID-19-confirmed patients were also high, especially 15 days after symptom onset. On the other hand, the positivity rates and sensitivities of the IgM assays (SuperFlex_IgM and iFlash_IgM) were lower than those of the pan-Ig assays, IgG assays, and combination assays in each period. At least in this study, there was no evidence that the IgM assays were superior to the pan-Ig or IgG assays. Therefore, pan-Ig assays, IgG assays and combination assays are all considered appropriate for both epidemiological studies and clinical use.

Although the usefulness of antibody testing for SARS-CoV-2 remains controversial, the CDC suggests its importance for public health and clinical use, e.g., for monitoring and responding to the COVID-19 pandemic [10]. They recommend choosing an assay with very high sensitivity and specificity. PerkinElmer SuperFlex™ contains 3 assays, pan-Ig, IgG, and IgM assays, and the pan-Ig and IgG assays have high sensitivities and remarkably high specificities. SuperFlex™ can also be used with the orthogonal testing algorithm. Therefore, SuperFlex™ is a feasible way to adhere to the CDC's recommendation.

Fifteen days after onset, pan-Ig assays, IgG assays and combination assays were positive, with a high positivity rate. On the other hand, in the early clinical stage, the positivity rate was low, and the concordance rate varied depending on the assay. These tendencies have also been observed in former studies [11–13]. In particular, SuperFlex_Ab and SuperFlex_any had significantly higher sensitivities than Elecsys—one of the FDA EUA assays. SuperFlex_Ab, SuperFlex_any, iFlash_IgG, and iFlash_any demonstrated the same performance in detecting SARS-CoV-2-specific antibodies and had relatively high sensitivity, even in the early stage. Increased sensitivity in the early stage is a great benefit for clinical diagnosis and serosurveillance. Although pan-Ig assays and IgG assays had good sensitivities relative to other than IgM assays, their performance in the early stage was suboptimal.

There remains no gold standard for COVID-19 antibody testing. One feasible strategy is to evaluate antibody assays using neutralizing antibodies as the standard. Of course, for the evaluation of convalescent plasma and vaccination, neutralizing assays should be used as the standard. However, for SARS-CoV-2-specific antibody testing assays, as used in this study, the sensitivity of antibody detection could be higher than that for neutralizing antibody testing for diagnosis. Moreover, the procedures are more complex for neutralizing assays than for antibody testing. Therefore, from a standpoint of seroprevalence, SARS-CoV-2-specific antibodies instead of neutralizing antibodies could be more easily detected and appropriate for study. Therefore, we defined seropositive as an antibody-positive result obtained from at least one assay among seroconversion-observed patients. However, this strategy may cause false-positive results. Each assay is a semiquantitative assay, and the antibody titer can increase. Even if only one positive result was obtained from one patient, the other assays could reveal a lower antibody titer increase that is below the cutoff level. In the early stage, between day 1 and day 14 after symptom onset, SuperFlex_Ab, SuperFlex_any, and iFlash_IgG had approximately 80% sensitivity. As described in a previous study, it is difficult to determine the optimal assay for the detection of SARS-CoV-2-specific antibodies during this period [11–13]. Fifteen days after symptom onset, the pan-Ig, IgG assays, and combination assays had remarkably high sensitivities, and no significant difference was found among them.

This study has several limitations, which should be acknowledged. First, the COG in this study included mainly COVID-19 patients hospitalized with severe illness and a small number of mild or asymptomatic patients. Some studies described negative results for antibody testing among patients who were asymptomatic or who had mild disease [14]. The patient characteristics widely differed, and the timing of blood sample collection could not be matched. Second, this study population included a relatively small number of patients at 56 days after onset. Further evaluation is needed to understand how long antibodies can be detected among COVID-19 patients. Third, a neutralizing antibody assay could not be performed. Therefore, it remains uncertain whether the antibody tests analyzed in this study can be correlated with that of neutralizing assays. Finally, the SARS-CoV-2 antibody assay that targets the spike protein cannot distinguish between antibodies derived from infection and vaccination. However, it remains valid for testing individuals who have not been vaccinated and for screening use for SARS-CoV-2 antibody detection. For instance, the SuperFlex™ assay can be performed easily and has a short turn-around time (15 min). Among the post-vaccinated cohort, antibody assays that target the nucleocapsid protein or envelope protein are appropriate for diagnosing COVID-19 infection.

In summary, within 14 days of symptom onset, the positivity rate of SARS-CoV-2 antibody testing was relatively low compared with 15 days after onset. During the late stage of infection, the sensitivities of pan-Ig, IgG and combination assays are very high. However, in the early stage, the sensitivities vary among the assays. The pan-Ig assays, the SuperFlex™ combination assay and the IgG and iFlash combination assays were more sensitive than the Elecsys pan-Ig assay approved under EUA by the FDA. For the diagnosis of COVID-19, antibody testing should be performed 15 days after onset. For epidemiological surveillance, assays with high sensitivity, even if in the early stage, should be used, such as SuperFlex_Ab, iFlash_IgG, and their combination. IgM assays were not suitable for these purposes.

## Data Availability

All data supporting the conclusions of this article are included in this article.
